# Effects of gallic acid on intraperitoneal adhesion bands in rats

**DOI:** 10.22038/AJP.2022.19811

**Published:** 2022

**Authors:** Mahmoud Hashemzaei, Kaveh Tabrizian, Mohammad Javad Koohkan, Mehdi Khoshsima Shahraki, Ramin Rezaee, Vahideh Ghorani, Jafar Shahraki

**Affiliations:** 1 *Department of Pharmacology and Toxicology, Faculty of Pharmacy, Zabol University of Medical Sciences, Zabol, Iran*; 2 *Student Research Committee, Faculty of Pharmacy, Zabol University of Medical Sciences, Zabol, Iran*; 3 *Toxicology and Addiction Research Center, Zabol University of Medical Sciences, Zabol, Iran*; 4 *Department of Pathology, Faculty of Medicine, Zabol University of Medical Sciences, Zabol, Iran*; 5 *International UNESCO Center for Health-Related Basic Sciences and Human Nutrition, Faculty of Medicine, Mashhad University of Medical Sciences, Mashhad, Iran*; 6 *Applied Biomedical Research Center, Mashhad University of Medical Sciences, Mashhad, Iran*; † * Equal first author*

**Keywords:** Gallic acid, Adhesion bands, TNF-α, Lipid peroxidation

## Abstract

**Objective:**

Gallic acid (GA) is an organic acid that possesses anti-inflammatory effects as it inhibits the production of metalloproteinases, tissue plasminogen activator, growth factors and adhesion molecules. Since formation of abdominal surgery-induced adhesion bands is accompanied by inflammation, angiogenesis and cell proliferation, in the current study, we assessed potential beneficial properties of GA against adhesion bands formation in rats.

**Materials and Methods:**

Thirty-six adult male rats were assigned into six groups of six animals. After induction of anesthesia, peritoneal injury was induced using a standard method and animals received either GA (10, 25, 50 and 100 mg/kg), or normal saline, while a group of rats remained intact. Seven days after the surgery, animals were decapitated and samples were collected for pathology evaluations. Also, lipid peroxidation (TBARS) and tumor necrosis factor alpha (TNF-α) levels were determined in serum samples.

**Results:**

Our results showed that GA significantly reduced lipid peroxidation in serum samples but had no effect on TNF-α levels. Furthermore, microscopic and macroscopic injuries reduced significantly in GA-treated animals.

**Conclusion:**

Since GA reduced adhesion bands formation at microscopic and macroscopic levels, it could be considered a treatment against adhesion bands formation.

## Introduction

Intraabdominal adhesion formation following gastrointestinal surgery is a very 

common consequence which induces intestinal obstruction, primary and secondary infertility, and pelvic pain, and raises healthcare costs. It is responsible for 74% intestinal obstruction, 20-50% cases of chronic pelvic pain and 15-20% of female infertility (Dubuisson et al., 2010 ▶). Although post-surgery adhesions are frequently experienced following upper and lower abdominal surgery, no definite recommendation has been proven to prevent these consequences. Also, commercially available products have not been approved for this purpose (Ouaïssi et al., 2012 ▶).

Following peritoneum injury, activation of mesothelium and endothelium leads to secretion of inflammatory cytokines (e.g. tumor necrosis factor alpha (TNF-α), interleukin 6 (IL-6), and IL-1) as well as growth factors such as vascular endothelial growth factors (VEGF) and adhesive proteins in the peritoneal cavity (Mohammadpour et al., 2015 ▶; Ouaïssi et al., 2012 ▶; Whang et al., 2011 ▶). The secretion of inflammatory cytokines such as TNF-α by activated macrophages, and growth factors is increased during early phase of wound healing (Cheong et al., 2002 ▶; Mohammadpour et al., 2015 ▶). Following injury, activated macrophages, neutrophils, macrophages and eosinophils are recruited to the location, and fibrinous exudates are released. Also, during injury, oxidative stress and lipid peroxidation occur and lead to formation of nascent exudates (Cheong et al., 2002 ▶; Liakakos et al., 2001 ▶). Moreover, cyclooxygenase (COX) (especially COX2) induction enhances the levels of pro-inflammatory cytokines like TNF-α, IL-1 and IL-6 that worsen the inflammation and promote the adhesion formation. Altogether, this process leads to activation of pro-inflammatory cytokines as well as growth factors and adhesive protein, exacerbating inflammation and adhesion band formation (Brochhausen et al., 2012 ▶; Parsaei et al., 2013 ▶). 

**Figure 1:**
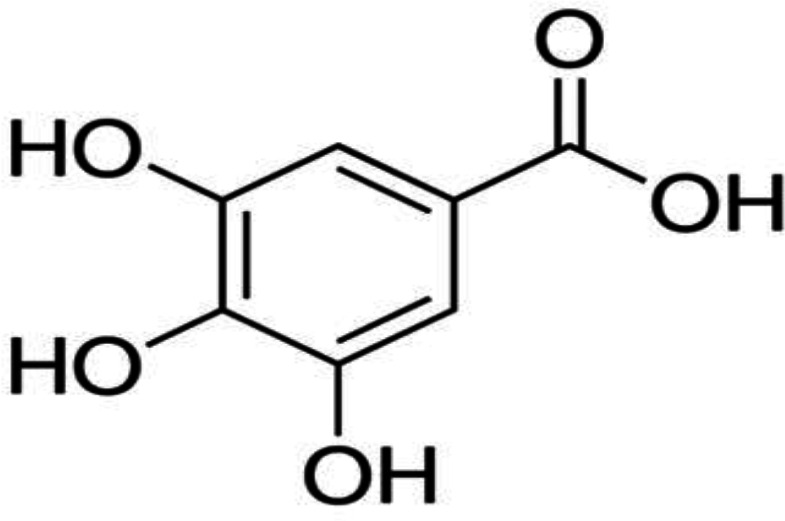
Chemical structure of gallic acid

Numerous natural products have been shown to have antioxidant and anti-inflammatory properties (Hashemzaei et al., 2016 ▶; Hashemzaei et al., 2017b ▶; Hashemzaei et al., 2020 ▶). Gallic acid (GA) is an organic acid (Figure 1) that exerts its anti-inflammatory effects via inhibition of pro-inflammatory cytokines production (Choi et al., 2009 ▶; Hsiang et al., 2013 ▶; Kim et al., 2006 ▶; Kroes et al., 1992 ▶). Furthermore, it can reduce the production of VEGF, IL-1, IL-6 and TNF-α, which are crucial for angiogenesis and adhesion band formation (Choi et al., 2009 ▶; Hsiang et al., 2013 ▶; Kim et al., 2006 ▶; Kroes et al., 1992 ▶). GA can suppress nitric oxide (NO) and prostaglandin E2 (PGE2) synthesis by repressing inducible nitric oxide synthase (iNOS) and COX2 (Hsiang et al., 2013 ▶; Moradi et al., 2020 ▶). It was confirmed that GA could reduce diclofenac-induced renal injury by antioxidant and anti-nitrosative effects (Moradi et al., 2020 ▶). It has been exhibited that GA has anti-inflammatory effects in lipopolysaccharide-induced macrophages in animals (Hsiang et al., 2013 ▶; Yilmaz et al., 2005 ▶).

Since various substances that are being used to reduce adhesion formation do not sufficiently diminish adhesion formation or have considerable side effects (Jomezadeh et al., 2012 ▶; Mohammadpour et al., 2015 ▶), we evaluated the effects of GA on the abdominal adhesion band formation following surgery in rats.

## Materials and Methods

**Materials**

Gallic acid and TNF-α kit were obtained from Sigma Aldrich (USA) and RayBiotech Co (USA), respectively.

**Animals**

In this study, 36 male Wistar rats (aging 9-10 weeks and weighing 200-250 g) were used. Animals were housed under normal conditions with 12hr/12hr dark/light cycles. Rats had free access to food and water. All animal experiments were performed with respect to Helsinki guidance and approved by the Ethics Committee of Zabol University of Medical Sciences, Zabol, Iran (ethics code No: Zbmu.1.rec.1396.138).

**Surgical procedure, treatments and histopathological/biochemical assessments**

**Figure 2:**
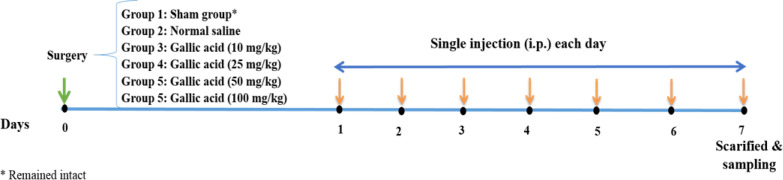
A schematic presentation of the study protocol

Animals underwent surgical procedures following induction of anesthesia by intraperitoneal (IP) administration of ketamine (100 mg/kg) and xylazine (10 mg/kg). The site of surgery was shaved and cleansed using alcohol and iodine solution. Then, a 3-cm midline laparotomy was done to access the abdominal cavity. The peritoneal button creation (PBC) technique as the most consistent and reproducible procedure was used to induce adhesion (Whang et al., 2011 ▶). In order to stimulate the formation of four peritoneal buttons, barbed 2/0 polypropylene sutures in a chain alignment were utilized. Each suture covered approximately 2 cm of parietal peritoneum and the tied sutures were approximately 5 mm in diameter. Animals were randomly assigned to 6 groups, including (1) sham group (group of rats that remained intact), (2) normal saline (NS) group (rats that received NS and were also called control group), and (3-6) four groups of GA-treated rats that intraperitoneally received GA at doses of 10, 25, 50 and 100 mg /kg (Kilic et al., 2019; Wei et al., 2018). Animals were housed in the recovery room for seven days, and they received a single injection each day. On day 7, rats were anaesthetized and sacrificed. Afterwards, animals underwent laparotomy, and peritoneal samples were collected for histopathological studies. Also, serum lipid peroxidation and TNF-α levels were evaluated. Duration of the study, injections and sampling are shown in Figure 2.

**Statistical analysis**

The data are presented as mean±SD. One-way analysis of variance (ANOVA) with *post-hoc* Tukey test was performed, using GraphPad Prism 6 for data analysis. P-values less than 0.05 were considered significant.

## Results

**Macroscopic adhesion intensity assessment**

**Figure 3:**
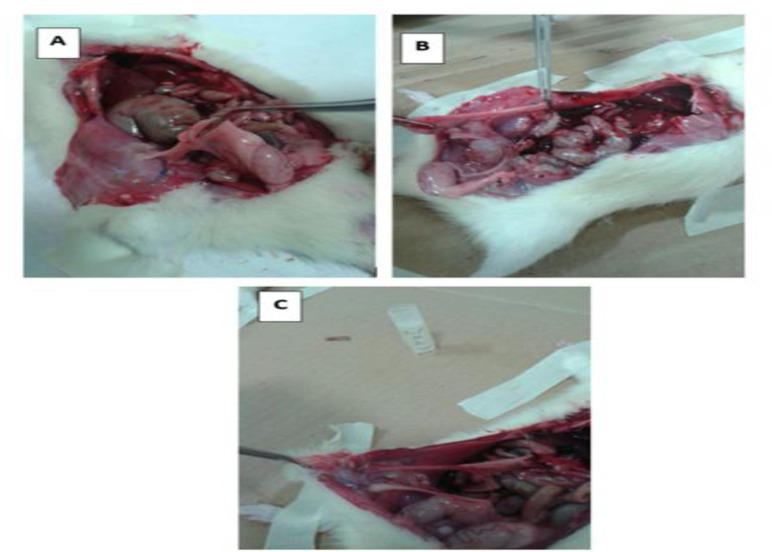
Macroscopic adhesion band scores: (A) thin bunches of a cellular fibrosis grade (score 1), (B) two areas of fibrosis (score 2) and (C) more than two areas of fibrosis (score 3)

According to the Nair scoring system (Nair et al., 1974), macroscopic adhesion was scored 1-3 as follows: (1) thin bunches of a cellular fibrosis, (2) two areas of fibrosis and (3) more than two areas of fibrosis (Figure 3). Our data indicated that severity and frequency of adhesion were decreased in the GA-treated groups in comparison to those treated with NS (i.e. the control group) (Table 1).

**Table 1:** The effects of GA on macroscopic peritoneal adhesions

Group	Score 1: Thin bunches of a cellular fibrosis	Score 2: Two areas of fibrosis	Score 3: Over two areas of fibrosis
NS	0/6	0/6	5/6
GA 10 mg/kg	1/6	2/6	3/6
GA 25 mg/kg	4/6	2/6	0/6
GA 50 mg/kg	4/6	2/6	0/6
GA 100 mg/kg	5/6	1/6	0/6

Table 1 present the numbers of animals (frequency) showing different adhesion severity scores following treatment with GA (10, 25, 50 and 100 mg/kg) and/or NS. The intensity of insults was categorized as three different scores including (1) thin bunches of a cellular fibrosis, (2) two areas of fibrosis and (3) more than two areas of fibrosis

**Evaluation of microscopic adhesion intensity**

Microscopic adhesions were studied by a pathologist focusing on inflammation, fibrosis and neovascularization. Concerning insults intensity, the microscopic insults were classified into four groups based on 0-3 scores defined as follows: (0) no microscopic insults, (1) thin bunches of cellular fibrosis, (2) wide areas of fibrosis with reduced vascularization and (3) areas of fibrosis formed by thick bunches of collagen (Yilmaz et al., 2005 ▶). Microscopic adhesion was diminished by GA in comparison to the NS-treated group (Table 2).

**Table 2:** The effects of GA on microscopic peritoneal adhesions

Group	Score 1: Thin bunches of a cellular fibrosis	Score 2: Wide areas of fibrosis with reduced fibrosis	Score 3: Areas of fibrosis formed by thick bunch of collagens
NS	0/6	1/6	3/6
GA 10 mg/kg	0/6	3/6	1/6
GA 25 mg/kg	1/6	3/6	0/6
GA 50 mg/kg	3/6	0/6	0/6
GA 100 mg/kg	2/6	2/6	0/6

Table 2 present the numbers of animals (frequency) showing different microscopic adhesion scores following treatment with GA (10, 25, 50 and 100 mg/kg) and/or NS. The intensity of insults was categorized as four different scores including (0) no fibrosis, (1) thin bunches of a cellular fibrosis, (2) wide areas of fibrosis with reduced fibrosis and (3) areas of fibrosis formed by thick bunch of collagens. According to the results, in none of the groups, score 0was observed.

**TBARS levels determination**

**Figure 4:**
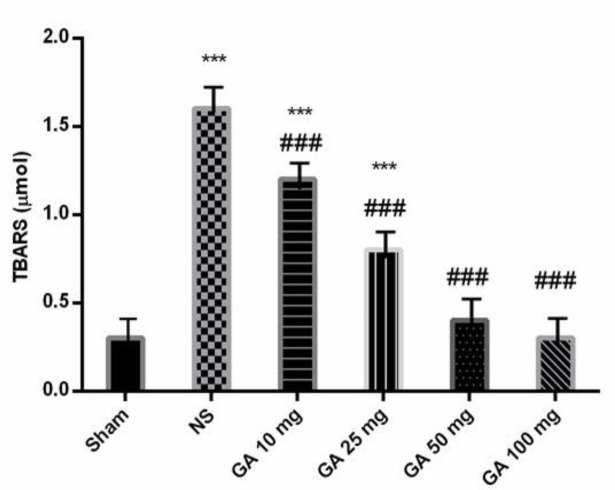
The effects of GA (10, 25, 50 and 100 mg/kg) on TBARS levels in rats (***p<0.001 in comparison to the sham-operated group and ###p<0.001 in comparison to the normal saline (NS)-treated group). TBARS: Thiobarbituric acid reactive substances

Following treatment with GA (10, 25, 50 and 100 mg/kg), thiobarbituric acid reactive substances (TBARS) level was significantly decreased in comparison to the NS-treated group (p<0.001; Figure 4). 

**Serum levels of TNF-α**

**Figure 5:**
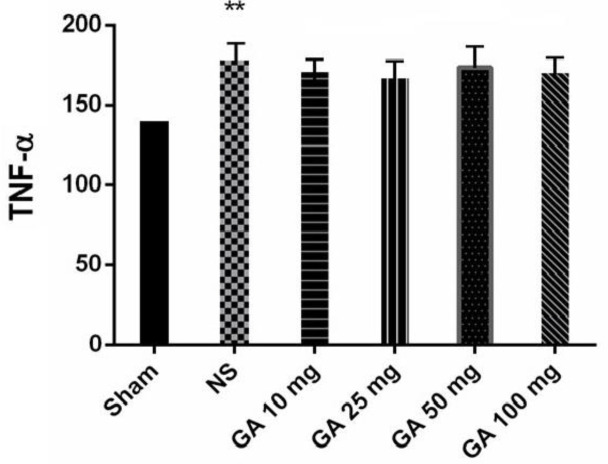
Serum levels of TNF-α (pg/ml) in different groups of rats (**p<0.01 in comparison to the sham-operated group).

Blood samples were collected seven days after the start of the study. The results showed that in the NS-treated group, serum levels of TNF- α were significantly increased compared to the sham group (p<0.01). Nevertheless, GA did not affect TNF-α level (Figure 5).

## Discussion

Abdominal surgery may cause adhesion formation which has clinical, economical and legal consequences; so far, no definite approach has been approved for prevention of adhesion formation. To fill this gap, numerous compounds including natural products and drugs with anti-inflammatory, anti-oxidant and wound-healing activities have been examined to prevent formation of adhesion bands (Hashemzaei et al., 2017a ▶; Jomezadeh et al., 2012 ▶; Mohammadpour et al., 2015 ▶). In the current study, the effects of GA in prevention of adhesion formation were evaluated in rats. Our results showed that GA could decrease adhesion formation at macroscopic and microscopic levels and reduce lipid peroxidation but did not affect serum levels of TNF-α. 

After peritoneal mesothelium dilation that occurs following inflammation, percolation of fibrinous exudates is observed outside the vessel. This fibrous tissue is composed of fibroblasts and extracellular matrix that eventually results in scar formation (Eckes et al., 2010 ▶). Furthermore, the presence of cytokines and other inflammatory mediators including TNF-α, IL-1, IL-6 and plasminogen results in macrophages invasion to the site leading to the adhesion of peritoneal site (Vykoukal and Davies, 2011 ▶).

Many substances like reactive oxygen species (ROS) scavengers (Binda et al., 2003 ▶), COX inhibitors (Guvenal et al., 2001 ▶), TNF-α antagonists (Kaidi et al., 1995 ▶), statins (Aarons et al., 2007 ▶) and hyaluronate/carboxymethyl cellulose (Binda et al., 2003 ▶; Demirbag et al., 2005 ▶) that can alleviate inflammatory reactions, have been examined to inhibit this process. 

In several studies, it was cleared that natural products such as curcumin (Jomezadeh et al., 2012 ▶), resveratrol (Üstün et al., 2007 ▶) and berberine (Zhang et al., 2014 ▶) can be used to reduce adhesion formation. Natural products that are found in our diet seem to have lesser adverse effects. GA is one of these compounds with diverse pharmacological effects; for instance, GA can suppress cytokines production and histamine release, and exerts ROS scavenging and COX2 inhibitory effects (Hassani et al., 2015 ▶; Inoue et al., 1994 ▶; Kroes et al., 1992 ▶). It was shown that GA boosts the antioxidant defense as it scavenges superoxide anions, and inhibits the release of myeloperoxidases. In this regard, the presence of o-dihydroxy moiety is essential for GA antioxidant properties (Devasagayam and Sainis, 2002 ▶; Pourmorad et al., 2006 ▶). In addition, GA is able to decrease inflammation through iNOS and COX2 inhibition (Hsiang et al., 2013 ▶). 

Matrix metalloproteinases (MMPs) particularly MMP-2 and MMP-9, plasminogen activator (PA) especially tissue-type PA(t- PA) induce the breakdown of the extracellular matrix and allow the cells to move from the tunica media into the intima, resulting in neo intimal hyperplasia and adhesion formation (Newby, 2006 ▶; Newby and Zaltsman, 2000 ▶; Vykoukal and Davies, 2011 ▶). GA can inhibit the production of PA, MMPs and t-PA (Hsiang et al., 2013 ▶). Furthermore, GA has anti-adhesive effects mediated via inhibition of growth factors and adhesive molecules such as VEGF, platelet-derived growth factor (PDGF) and vascular cell adhesion molecule-1 (VCAM-1) (Hsiang et al., 2013 ▶). 

VEGF is a key protein with strong vasculogenesis and angiogenesis properties that can facilitate adhesion formation via angiogenesis (Yoshida et al., 1996 ▶). It was revealed that VEGF antibodies inhibit adhesion formation by inhibition of angiogenesis and vasculogenesis (Cahill and Redmond, 2008 ▶; Diamond et al., 2005 ▶; Saltzman et al., 1996 ▶). GA inhibits the production of VEGF, angiogenesis and vasculogenesis that are necessary for producing and expanding adhesion bands (Hsiang et al., 2013 ▶).

Lipid peroxidation induces adhesion formation (Ara et al., 2005 ▶; Heydrick et al., 2007 ▶; Özçelik et al., 2003 ▶). Melatonin is one of the most powerful free-radical scavengers that were used against postoperative adhesion formation and it could significantly reduce adhesion formation and reduce pro-inflammatory cytokines (Ara et al., 2005 ▶; Heydrick et al., 2007 ▶; Özçelik et al., 2003 ▶). Furthermore, methylene blue, a highly redox active dye, could reduce adhesion formation in animals (Heydrick et al., 2007 ▶). Our results are consistent with those of the aforementioned studies, confirming that GA reduces oxidative stress and decreases adhesion formation.

The present results did not show any significant decrement in serum TNF-α levels following GA treatment. However, as a limitation of the present study, TNF-α levels were only assessed in serum and determination of its levels in tissues should be considered by future studies. 

In conclusion, the results confirmed that GA can inhibit adhesion formation after surgery in rats. In the current study, we showed that GA reduced microscopic and macroscopic adhesion and decreased lipid peroxidation but had no effects on serum levels of TNF-α. Hence, we suggest that GA could be considered a treatment against adhesion bands formation.

## Conflicts of interest

The authors have declared that there is no conflict of interest.
